# Association of estimated pulse wave velocity with cardiovascular disease outcomes and all-cause death—a systematic review and meta-analysis

**DOI:** 10.3389/fcvm.2025.1641697

**Published:** 2025-09-16

**Authors:** Jian Li, Fa Gao, Fang Cao, Shan Lv, Yulong Hou, Wei Guo, Chongheng Zhang, Aidong Liu

**Affiliations:** ^1^ College of Chinese Medicine, Changchun University of Chinese Medicine, Changchun Jilin, China; ^2^ School of Basic Medicine, Changchun University of Chinese Medicine, Changchun Jilin, China; ^3^ Department of Cardiology, The Affiliated Hospital of Changchun University of Chinese Medicine, Changchun Jilin, China; ^4^ Department of Cardiology, The Third Affiliated Hospital of Changchun University of Chinese Medicine, Changchun Jilin, China

**Keywords:** estimated pulse wave velocity, cardiovascular risk, cardiovascular disease, mortality prediction, meta-analysis, arterial stiffness

## Abstract

**Background:**

**:** The estimated pulse wave velocity (ePWV), derived from age and mean blood pressure (MBP) in accordance with the Reference Values of Arterial Stiffness Collaboration, has emerged as a novel alternative indicator for assessing arterial stiffness. This systematic review and meta-analysis aims to assess the correlation of ePWV with the likelihood of adverse cardiovascular (CV) events and all-cause mortality.

**Methods:**

Studies published before February 2024 from PubMed, Embase, Cochrane Library, and Web of Science were searched. To ensure the completeness and timeliness of the included literature, a thorough re-search and update of the relevant literature were conducted on April 28, 2025. The data analysis was carried out utilizing STATA (V15.0).

**Results:**

A systematic review and meta-analysis of 20 studies involving 381,303 participants demonstrated that individuals with higher ePWV had significantly increased risks of total CV events (HR = 2.14, 95%CI: 1.70–2.71), CV mortality (HR = 3.64, 95%CI: 2.83–4.68), and all-cause mortality (HR = 1.85, 95%CI: 1.38–2.47). Specifically, for each 1 m/s increase in ePWV, the risks of these outcomes increased by 36%, 41%, and 37%, respectively. Analyses of population types further verified that elevated ePWV was independently associated with increased risks for all outcomes. For total CV events, the HRs were 1.79 (95%CI: 1.45–2.21) in the general population and 3.43 (95%CI: 2.62–4.49) in those with CVD. For CV mortality, the HRs were 4.90 (95%CI: 2.78–8.64) and 3.39 (95%CI: 2.56–4.49), respectively. For all-cause mortality, HRs were 2.28 (95%CI: 1.00–5.21) in the general population and 1.84 (95%CI: 1.20–1.42) in the CVD group. Moreover, each 1 m/s increase in ePWV was associated with a 27% and 54% increase in total CV event risk, a 28% and 54% increase in CV mortality, and a 47% and 30% increase in all-cause mortality in the general and CV populations, respectively.

**Conclusion:**

These findings highlight ePWV as a potential predictor of adverse health outcomes, warranting further research to establish reference values and compare with carotid-femoral pulse wave velocity.

**Systematic Review Registration:**

PROSPERO CRD42024536235.

## Introduction

Cardiovascular disease (CVD) is an important noncommunicable disease that lead to the increase in global mortality and incidence (1). As of 2019, the total number of CVD cases has surged to 523 million, showing an increase by 252 million compared to 1990. The death toll has elevated from 12.1 million to 18.6 million (2), bringing huge health and economic burdens to the world. The incidence of CVD in underdeveloped and low-income countries has increased significantly, accounting for about 80% (3). The occurrence of CVD is closely related to hypertension, hyperlipidemia, smoking, drinking, and other risk factors. Arterial stiffness caused by multiple risk factors is a sign of vascular aging. Therefore, arterial stiffness, a prevalent metric for assessing vascular aging, also stands as a marked risk factor for CVD. A rise in arterial stiffness will diminish the elasticity and buffering capacity of the arterial wall, and increase the stress and blood flow fluctuations on the vessel wall, leading to cardiovascular (CV) dysfunction (4). It has demonstrated that (5) changes in arterial stiffness may occur before the obvious manifestations of CVD. Therefore, early assessment of arterial stiffness is particularly important for the long-term prognosis of CVD. Arterial stiffness, serving as a standalone indicator of CV risk, is widely used in clinical evaluation of patients and has been identified as a robust predictor of CV events and all-cause death (6–8). Carotid-femoral pulse wave velocity (cfPWV) is a golden indicator that directly reflects aortic stiffness (9) and has the best predictive value in the assessment of the risk of total CV events (10, 11). Brachial-ankle pulse wave velocity (baPWV) is also a measure of arterial health. Compared to cfPWV, baPWV offers the advantages of a simpler procedure and easier access to measurement sites. Nonetheless, it still relies on specialized medical equipment. baPWV measures pulse wave velocity from the brachial artery to the ankle artery. Its extended path length and broader vascular territory render baPWV more appropriate for a comprehensive assessment of systemic arterial health. However, in evaluating aortic stiffness and CV risk, the accuracy of cfPWV remains relatively higher (12). However, measuring cfPWV requires professional medical stuff to operate on specific equipment, which is expensive and not commonly found in clinical settings. This limitation particularly affects countries and regions with limited medical resources, where widespread implementation is challenging, thus restricting access to relevant indices (9, 13). In addition, the surface distance between the measurement locations of the carotid and femoral arteries may not accurately represent the actual arterial path length. Therefore, there is an urgent need for an optimized and simplified prediction method to help advance research on arteriosclerosis and its consequences.

Currently, estimated pulse wave velocity (ePWV) derived from age and mean blood pressure (MBP) is suggested as a substitute for cfPWV (14). Age and blood pressure, as two important clinical parameters of arteriosclerosis, are not only easy to collect, but also convenient for daily monitoring of the severity of aortic stiffness (15, 16). ePWV has been proven to have highly consistent predictive value with cfPWV. It is worth noting that ePWV has the capacity to predict CV events regardless of traditional CV risk factors such as SCORE, FRS, BMI, and cfPWV. Many prospective cohort studies have also tested the role of ePWV in predicting future CVD event risk and all-cause death (17, 18). While multiple studies have showcased the predictive value of ePWV, a comprehensive quantitative assessment of its impact is currently lacking.

In recent times, there has been a growing research emphasis on examining the correlation of ePWV with the likelihood of CV events and all-cause mortality. Additionally, the research participants of ePWV prediction are relatively diverse, involving different regions and races, which may lead to different risk estimates and stratification. More importantly, it is important to understand whether the predictive ability of ePWV exceeds the scope of predictive events. Therefore, a thorough systematic review and meta-analysis were conducted utilizing the existing body of evidence.

## Methods

This meta-analysis adhered to the PRISMA Statement 2020 guidelines (the latest guidelines for systematic review report) for reporting (19). The PRISMA checklist is provided in the Supplementary Table S1. The study protocol was registered on PROSPERO official website (registration No: CRD42024536235).

### Retrieval strategy

Relevant studies published in PubMed, Embase, Cochrane Library, and Web of Science were retrieved within the time period from database establishment to February 18, 2024. Considering the potential for ongoing updates within databases, a re-search of the relevant literature was conducted on April 28, 2025, to ensure the completeness and timeliness of the included literature. Searches were conducted using MeSH terms and free-text terms, including “pulse wave velocity”, “cardiovascular diseases”, “Mortality”, “carotid-femoral pulse wave velocity”, “ePWV”, “cardiocerebrovascular disease”, “estimate”, “all-cause mortality”, as well as all relevant terms. See the Supplementary Table S2 for the specific search strategy. Additionally, explorations of reference lists within pertinent articles were conducted to pinpoint eligible studies.

### Inclusion/exclusion criteria

The inclusion and exclusion criteria were established in this meta-analysis according to the PECOS principle, and the inclusion criteria were as follows: (1) Individuals aged 18 years and above, encompassing both the general population and those with cardiovascular conditions. (2) Studies that have evaluated ePWV (the formula published in Reference Values for Arterial Stiffness Collaboration in 2010 was applied for calculation. The ePWV of subjects with CV risk factors was calculated using formula one, while the ePWV of subjects without risk factors and who did not smoke was calculated using formula two. For detailed formulas, please refer to the Supplementary Appendix); (3) Articles that have explored the correlation of ePWV with major adverse CV events (CV death and nonfatal CV events), CV death, or all-cause death risk assessment; (4) Retrospective or prospective cohort studies; (5) Articles that are written in English.

The exclusion criteria were as follows: (1) Articles that did not meet the requirements, such as reviews, meta-analyses, guidelines, letters, conferences, replies, and abstracts; (2) Studies unobtainable in full text or duplicate published studies; (3) Studies with animal subjects; (4) Studies with exposure factors other than ePWV; (5) Studies with non-survival or CV events as the outcome; (6) The data are incomplete or unclear. If multiple studies were published using the same cohort, the study with a longer follow-up time or the largest sample size would be selected.

### Article screening and data extraction

Endnote X9 software was applied to screen for studies that should be included and excluded. The duplicate studies were first removed, and articles that did not meet the standards were initially screened out based on the title and abstract. The full texts were downloaded and read to evaluate whether the articles met the inclusion criteria. The final extracted data incorporated: the first author, date of publication, country and region, study design, sample size, age, study population, study duration, primary outcome, adjustment factors, quality evaluation, and other relevant characteristics. Two researchers (G.F. and C.F.) independently carried out the screening of articles and the extraction of data according to predetermined principles. Then, the data were checked and compared. If there are any discrepancies, the two parties will discuss to get a resolution. If the issue still cannot be resolved, it will be submitted to the third researcher (L.S.) for assistance in completing the final decision.

### Quality assessment

The Newcastle Ottawa Scale (NOS) (20). Tools were adopted to evaluate the selection, comparability, and quality of outcomes included in the study. The NOS scale comprised three modules encompassing a total of eight items. Specifically, four items were designated for study subject selection, one for group comparability, and three for outcome assessment. The overall score spanned from 0 to 9, with ratings falling into categories of low quality (0–3 points), medium quality (4–6 points), and high quality (7–9 points). Quality assessment tools are available in Supplementary Table S3.

### Statistical analysis

The analysis of all extracted data was performed utilizing Stata (V15.0). Meta-analysis was conducted by collecting OR, RR, or HR as effect measures for research reports. In each study, the effect values of high and low stiffness groups were calculated as categorical variables, and the effect values of each absolute ePWV difference (1 m/S and 1 SD) were calculated as continuous variables. If results adjusted for different covariates were reported in several included studies, the value adjusted for maximum covariates was extracted. All evaluation measures reported 95%CIs, and the findings of the analysis were visually depicted through a forest plot. The DerSimonian and Laird (DL) method was adopted to estimate the variance (*τ*^2^) between studies and assess the random-effects model. Cochran's *Q* test and *I*^2^ index were adopted to test the heterogeneity of the articles. In cases where heterogeneity was deemed non-significant, a fixed-effects model was employed for the pooled analysis; conversely, a random-effects model was chosen for pooling. Subgroup analyses and sensitivity analyses were conducted to explore the sources of heterogeneity observed in the results. A Jackknife sensitivity analysis was additionally carried out to evaluate the robustness of the results. Funnel plots were generated to evaluate the potential presence of publication bias in the included articles, and statistical testing for publication bias was conducted using Egger's method. Statistical significance was set at *P* < 0.1 (21, 22). If publication bias was found to be significant, the trim-and-fill method was utilized to evaluate its impact on bias in the observed outcomes. Except for Egger test, other results with *P* < 0.05 indicated statistical differences.

## Results

### Literature retrieval

**Figure 1:**
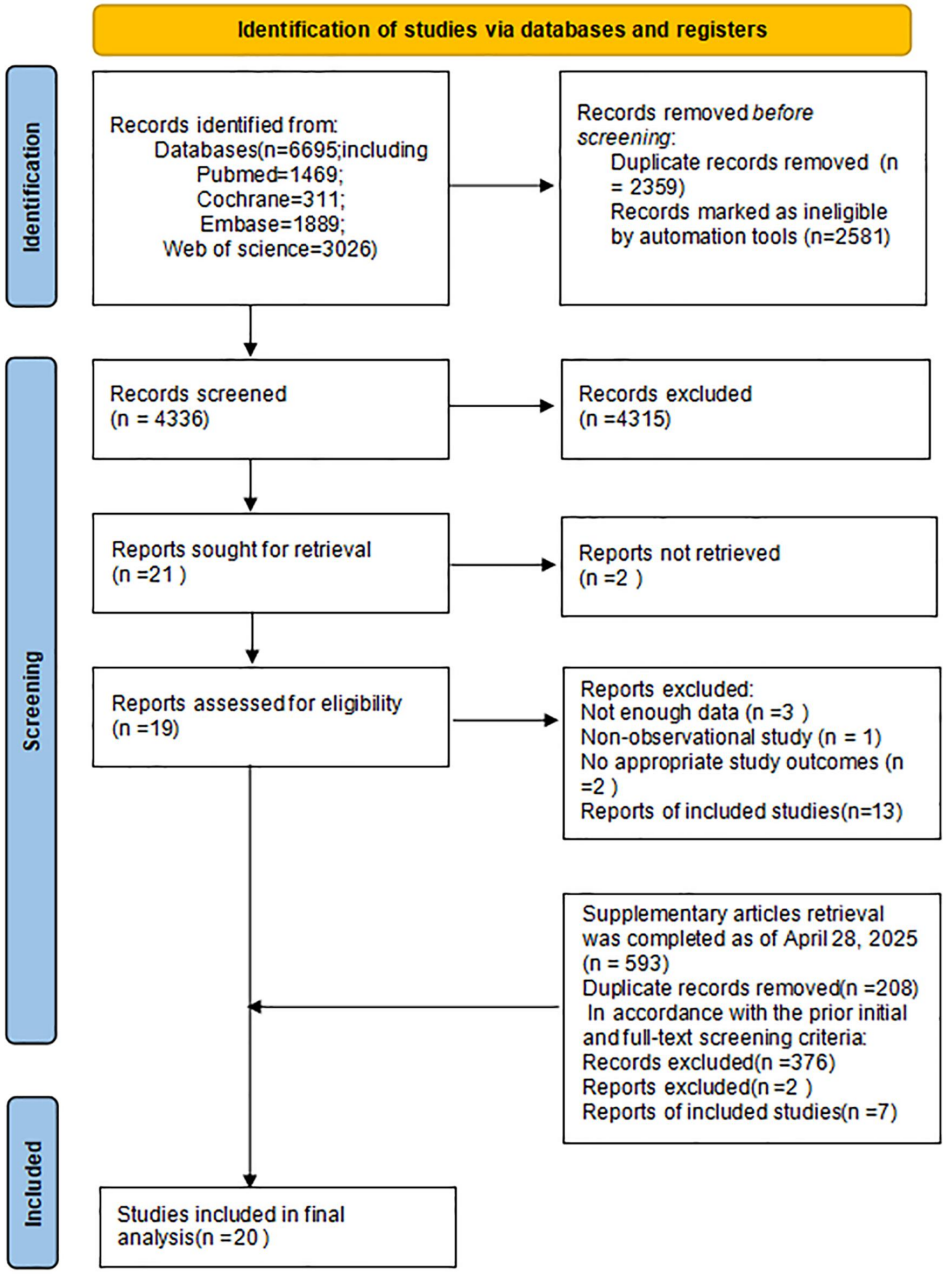
Flowchart of literature screening.

Altogether 6,695 studies were preliminarily retrieved according to the retrieval plan. Of them, 2,359 duplicate studies were excluded; and 4,308 were eliminated following the screening of titles and abstracts. Subsequently, the remaining 21 articles underwent full-text assessment. Based on the application of the inclusion and exclusion criteria, 13 studies were ultimately deemed eligible for inclusion. An additional search was conducted for literature published between January 19, 2024, and April 28, 2025, yielding 593 articles. After applying the same screening process, 7 new articles were identified that met the inclusion criteria. Ultimately, 20 studies were incorporated into this meta-analysis. The process of literature screening is illustrated in Figure 1.

### Characteristics and quality assessment of included articles

Supplementary Table S4 displays the fundamental attributes of the studies that were ultimately included. A total of 20 studies (14, 17, 18, 23–29) fulfilled the criteria for our meta-analysis. These articles were published between 2016 and 2025, and altogether 381,303 participants were analyzed in the included studies. Among these studies, 16 reported total CV events (14, 17, 18, 23–35) involving 340,062 individuals, 8 studies assessed CV mortality separately involving 183,022 individuals (17, 24, 26, 29–31, 33, 34) and 12 reported all-cause mortality outcomes involving 311,868 individuals (17, 18, 24, 29–31, 33, 34, 36–39). Among these studies, 12 were conducted in Asia (11 in China (17, 18, 28, 30–33, 35–38), 3 in South Korea (25–27), 6 in Europe (3 in Denmark (14, 24, 39), 1 in Germany (34), 1 in Croatia (29), and 1 in the Americas (the United States) (23). All the studies included in the analysis adjusted for potential confounding variables, including age, gender, drinking history, smoking history, BMI, CV and cerebrovascular history. Quality assessment using the NOS indicated that 3 studies (24, 35, 38) were rated as moderate quality, while 17 studies (14, 17, 18, 23, 24, 36, 37) were rated as high quality.

### Meta-analysis

A meta-analysis was conducted on different outcomes (total CV events, CV mortality, and all-cause mortality). The pooled HR of high ePWV and low ePWV was calculated. In addition, based on the linear relationship between ePWV and clinical events in the included studies, the pooled HR for every 1 m/S and 1 SD was also calculated. In addition, considering the significant differences between different population groups, separate analyses were conducted for each outcome by population type.

#### ePWV and total CV events

**Figure 2:**
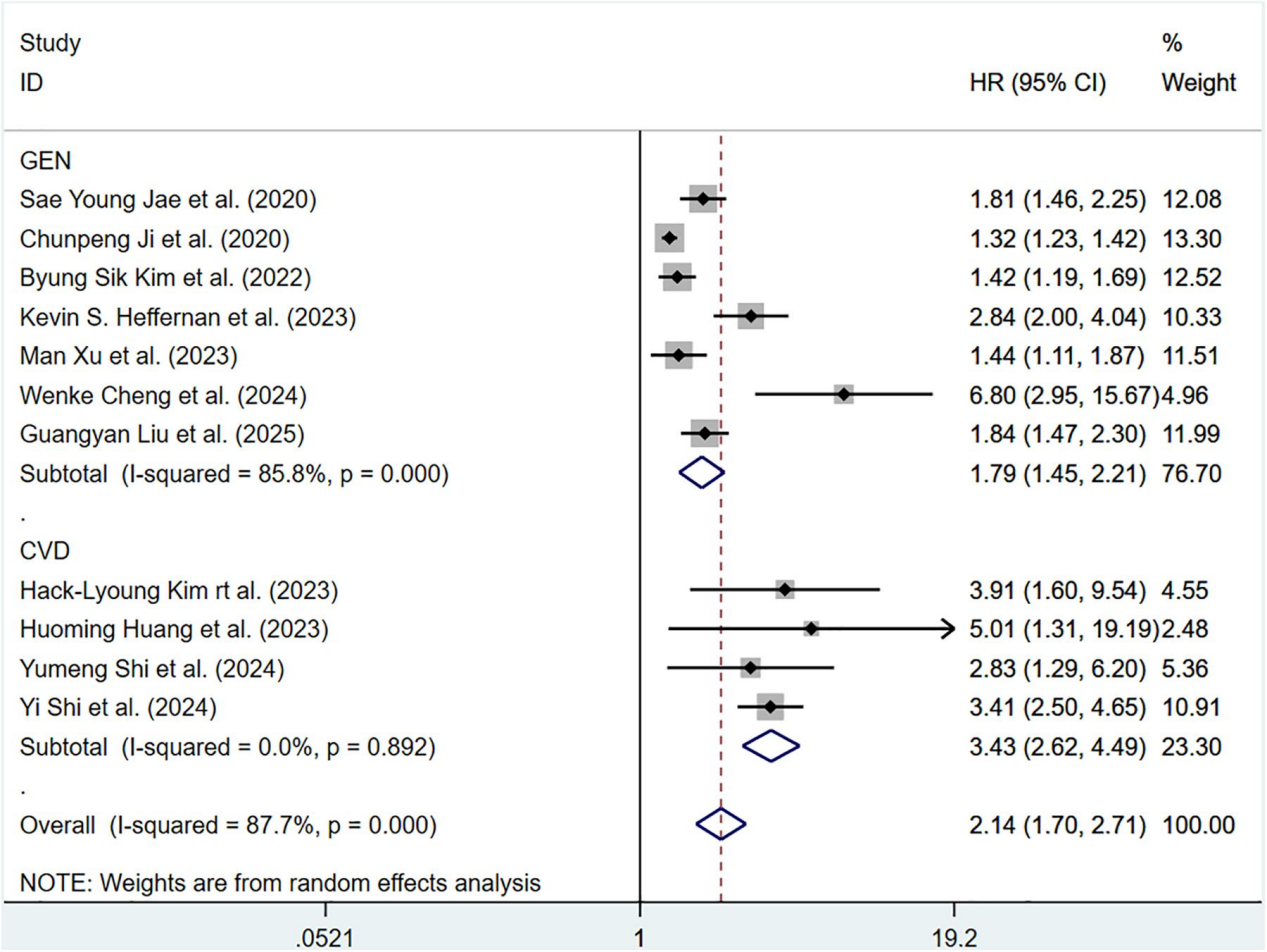
Subgroup analysis of CV events in general population and those with CVD, analyzed according to categorical variables. The results of each study are represented in point estimates (represented by black diamonds in the figure), and the horizontal line around each point estimate represents the 95% confidence interval (CI) of the research results. The width of the CI represents the accuracy of the estimated value; a narrower CI means higher accuracy. The gray square represents the effect size weight of a single study, and its area is proportional to the weight of the study in the meta-analysis. The hollow diamond at the bottom represents the summary effect size and its CI, which represents the final result of the meta-analysis. The coincidence point between the center position of the diamond and the red dashed line is the summary effect size. The horizontal axis (*X*-axis) represents the numerical range of the effect size. The logarithmic scale centered around 1 in the figure represents negative effect if it is less than 1, and positive effect if it is greater than 1. The black solid line crossing vertically through 1 represents “no effect” or “no difference”. If the CI of the study intersects with the invalid line, it indicates no statistical significance, otherwise it can be considered as significant differences.

Sixteen cohort studies involving 340,062 participants reported the association between ePWV and total CV events. Heterogeneity analysis showed considerable heterogeneity among the included articles (*P* < 0.001, *I*^2^ = 87.7%). Thus, a random-effects model was adopted to pool the results. Compared with individuals with low ePWV, individuals with high ePWV showed a significantly higher risk of total CV events (HR = 2.14, 95%CI: 1.70–2.71) (Supplementary Figure S1). Separate analyses were conducted based on different population types, and we found that individuals with high ePWV had significantly higher risk levels in both the general population group (HR: 1.79, 95%CI: 1.45–2.21, *P* < 0.001, *I*^2^ = 85.8%) and the CVD group (HR: 3.43, 95%CI: 2.62–4.49, *P* = 0.892, *I*^2^ = 0.0%) (Figure 2). In addition, this result revealed that population type is a potential source of heterogeneity. The non-general population (*I*^2^ = 0.0%) exhibited improved homogeneity. Further exploration of heterogeneity during follow-up years also revealed improved homogeneity when the follow-up duration was ≤5 years (*P* = 0.594, *I*^2^ = 0.0%) (Supplementary Table S5). Meta regression analysis of 10 studies showed that ePWV did not show significant correlation with the observed heterogeneity (*β* = 1.147, 95%CI: 0.871–1.512, *P* = 0.284) (Supplementary Figure S2).

**Figure 3:**
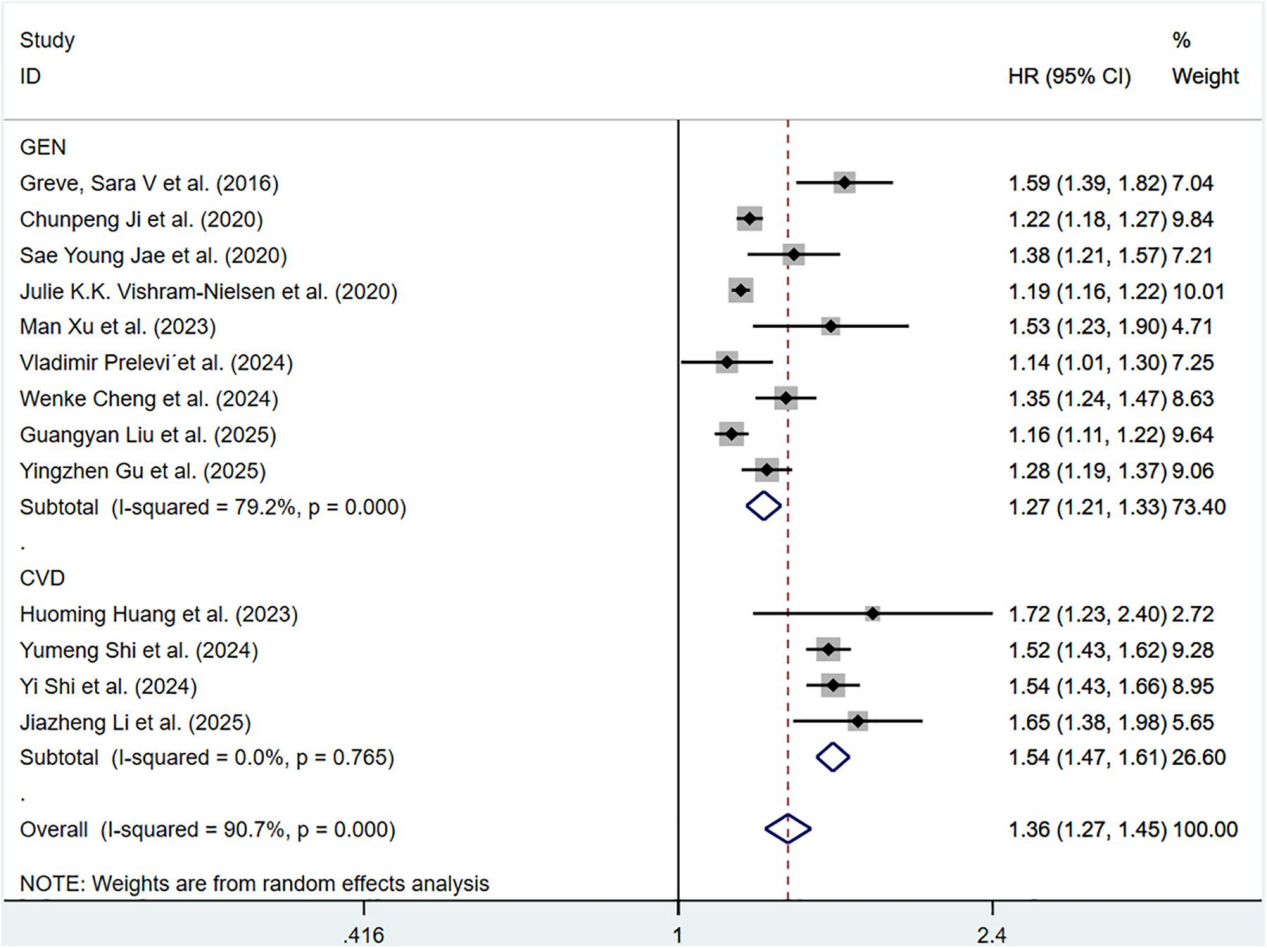
Subgroup analysis of CV events in general population and those with CVD population, analyzed according to continuous variables. The results of each study are represented in point estimates (represented by black diamonds in the figure), and the horizontal line around each point estimate represents the 95% confidence interval (CI) of the research results. The width of the CI represents the accuracy of the estimated value; a narrower CI means higher accuracy. The gray square represents the effect size weight of a single study, and its area is proportional to the weight of the study in the meta-analysis. The hollow diamond at the bottom represents the summary effect size and its CI, which represents the final result of the meta-analysis. The coincidence point between the center position of the diamond and the red dashed line is the summary effect size. The horizontal axis (*X*-axis) represents the numerical range of the effect size. The logarithmic scale centered around 1 in the figure represents negative effect if it is less than 1, and positive effect if it is greater than 1. The black solid line crossing vertically through 1 represents “no effect” or “no difference”. If the CI of the study intersects with the invalid line, it indicates no statistical significance, otherwise it can be considered as significant differences.

For continuous data, considerable heterogeneity was observed among the included articles (*P* < 0.001, *I*^2^ = 90.7%). Hence, a random-effects model was applied. The pooled HR of total CV events per 1 m/S and 1 SD increase in ePWV was 1.36 (95%CI: 1.27–1.45) (Supplementary Figure S3). Individuals with high ePWV had significantly higher risk levels in both the general population group (HR: 1.27, 95%CI: 1.21–1.33, *P* < 0.001, *I*^2^ = 79.2%) and the CVD group (HR: 1.54, 95%CI: 1.47–1.61, *P* = 0.765, *I*^2^ = 0.0%) (Figure 3). The non-general population (*I*^2^ = 0.0%) exhibited improved homogeneity.

#### ePWV and CV mortality

**Figure 4:**
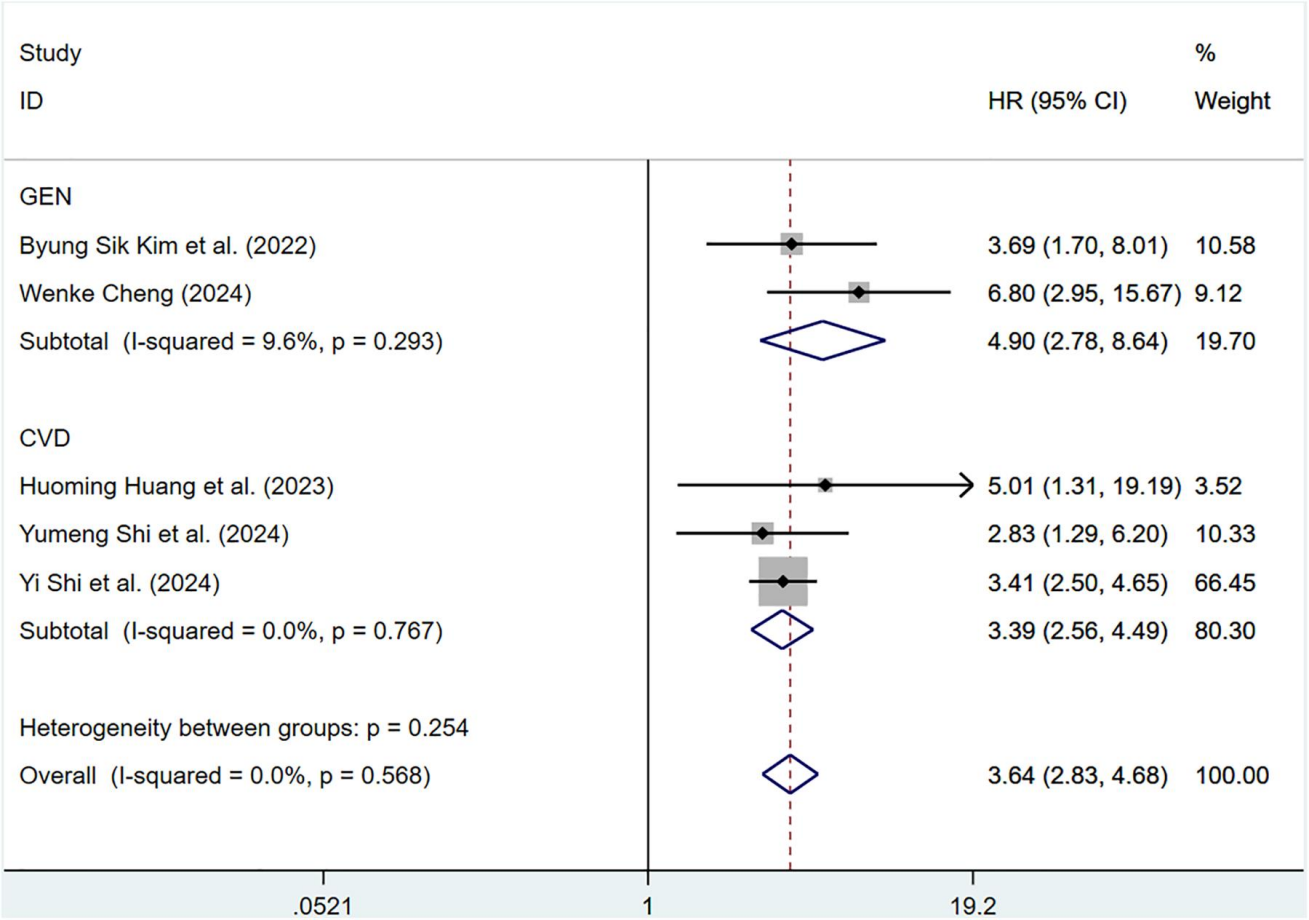
Subgroup analysis of CV mortality events in general population and those with CVD, analyzed according to categorical variables. The results of each study are represented in point estimates (represented by black diamonds in the figure), and the horizontal line around each point estimate represents the 95% confidence interval (CI) of the research results. The width of the CI represents the accuracy of the estimated value; a narrower CI means higher accuracy. The gray square represents the effect size weight of a single study, and its area is proportional to the weight of the study in the meta-analysis. The hollow diamond at the bottom represents the summary effect size and its CI, which represents the final result of the meta-analysis. The coincidence point between the center position of the diamond and the red dashed line is the summary effect size. The horizontal axis (*X*-axis) represents the numerical range of the effect size. The logarithmic scale centered around 1 in the figure represents negative effect if it is less than 1, and positive effect if it is greater than 1. The black solid line crossing vertically through 1 represents “no effect” or “no difference”. If the CI of the study intersects with the invalid line, it indicates no statistical significance, otherwise it can be considered as significant differences.

**Figure 5:**
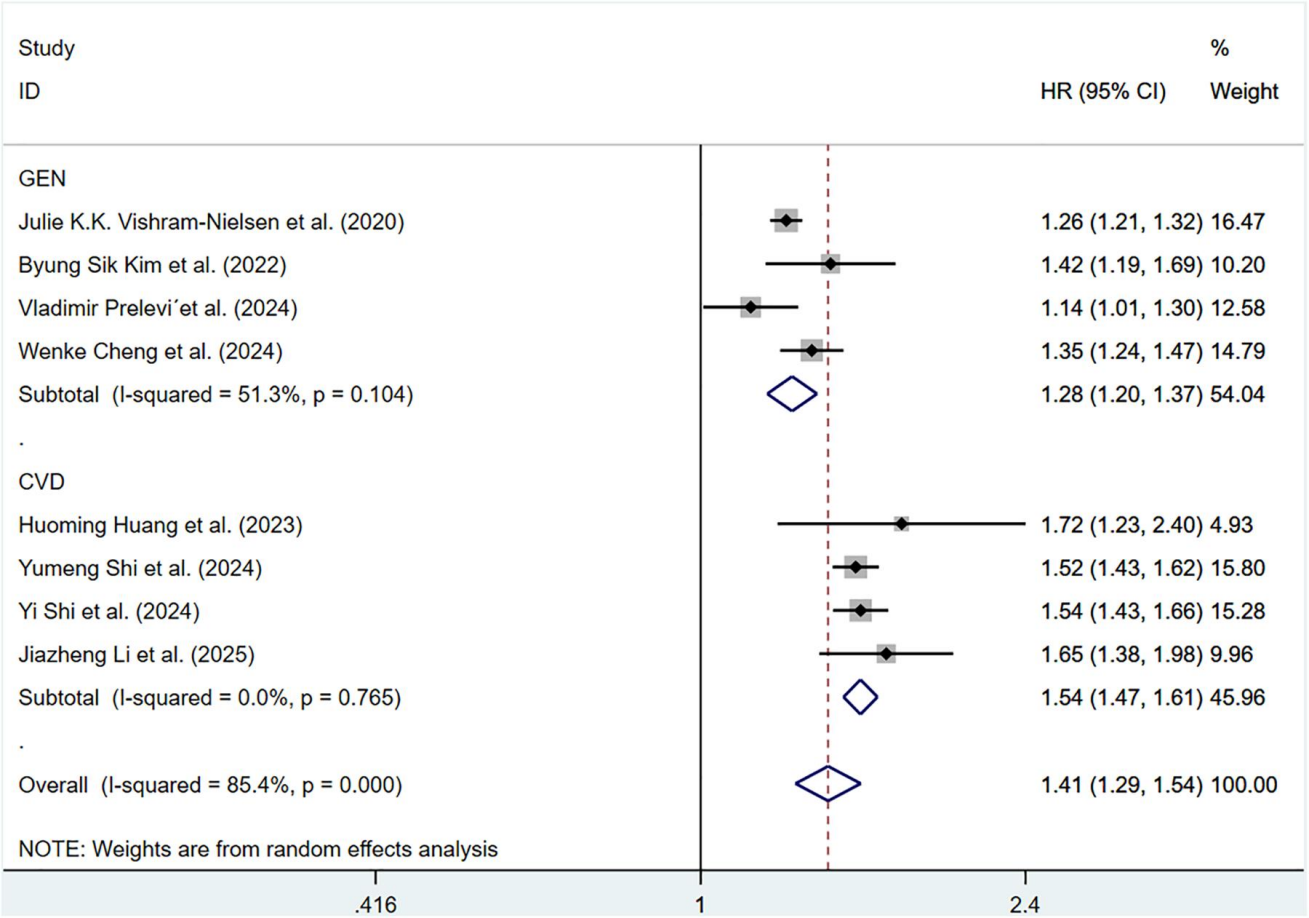
Subgroup analysis of CV mortality events in general population and those with CVD, analyzed according to continuous variables. The results of each study are represented in point estimates (represented by black diamonds in the figure), and the horizontal line around each point estimate represents the 95% confidence interval (CI) of the research results. The width of the CI represents the accuracy of the estimated value; a narrower CI means higher accuracy. The gray square represents the effect size weight of a single study, and its area is proportional to the weight of the study in the meta-analysis. The hollow diamond at the bottom represents the summary effect size and its CI, which represents the final result of the meta-analysis. The coincidence point between the center position of the diamond and the red dashed line is the summary effect size. The horizontal axis (*X*-axis) represents the numerical range of the effect size. The logarithmic scale centered around 1 in the figure represents negative effect if it is less than 1, and positive effect if it is greater than 1. The black solid line crossing vertically through 1 represents “no effect” or “no difference”. If the CI of the study intersects with the invalid line, it indicates no statistical significance, otherwise it can be considered as significant differences.

Eight cohort studies involving 183,022 participants discussed the correlation of ePWV with CV mortality. Compared with individuals with low ePWV, individuals with high ePWV showed a significantly higher risk of CV mortality (HR = 3.64, 95%CI, 2.83–4.68) (Supplementary Figure S4). Heterogeneity analysis showed the heterogeneity among the included articles was not important (*P* = 0.568, *I*^2^ = 0.0%). Hence, a fixed-effects model was adopted. Analyses of population types verified that individuals with high ePWV had significantly higher risk in both the general population group (HR: 4.90, 95%CI: 2.78–8.64, *P* = 0.293, *I*^2^ = 9.6%) and the CVD group (HR: 3.39, 95%CI: 2.56–4.49, *P* = 0.767, *I*^2^ = 0.0%) (Figure 4). The pooled HR of CV mortality with an elevation of 1 m/S and 1 SD in ePWV was 1.41 (95%CI: 1.29–1.54) (Supplementary Figure S5). Considerable heterogeneity was observed among the included articles (*P* < 0.001, *I*^2^ = 85.4%). Hence, a random-effects model was utilized. Individuals with high ePWV had significantly higher risk levels in both the general population group (HR: 1.28, 95%CI: 1.20–1.37, *P* = 0.104, *I*^2^ = 51.3%) and the CVD group (HR: 1.54, 95%CI: 1.47–1.61, *P* = 0.765, *I*^2^ = 0.0%) (Figure 5). The population type remained a potential source of heterogeneity, and the non-general population (*I*^2^ = 0.0%) exhibited improved homogeneity.

#### ePWV and all-cause mortality

**Figure 6:**
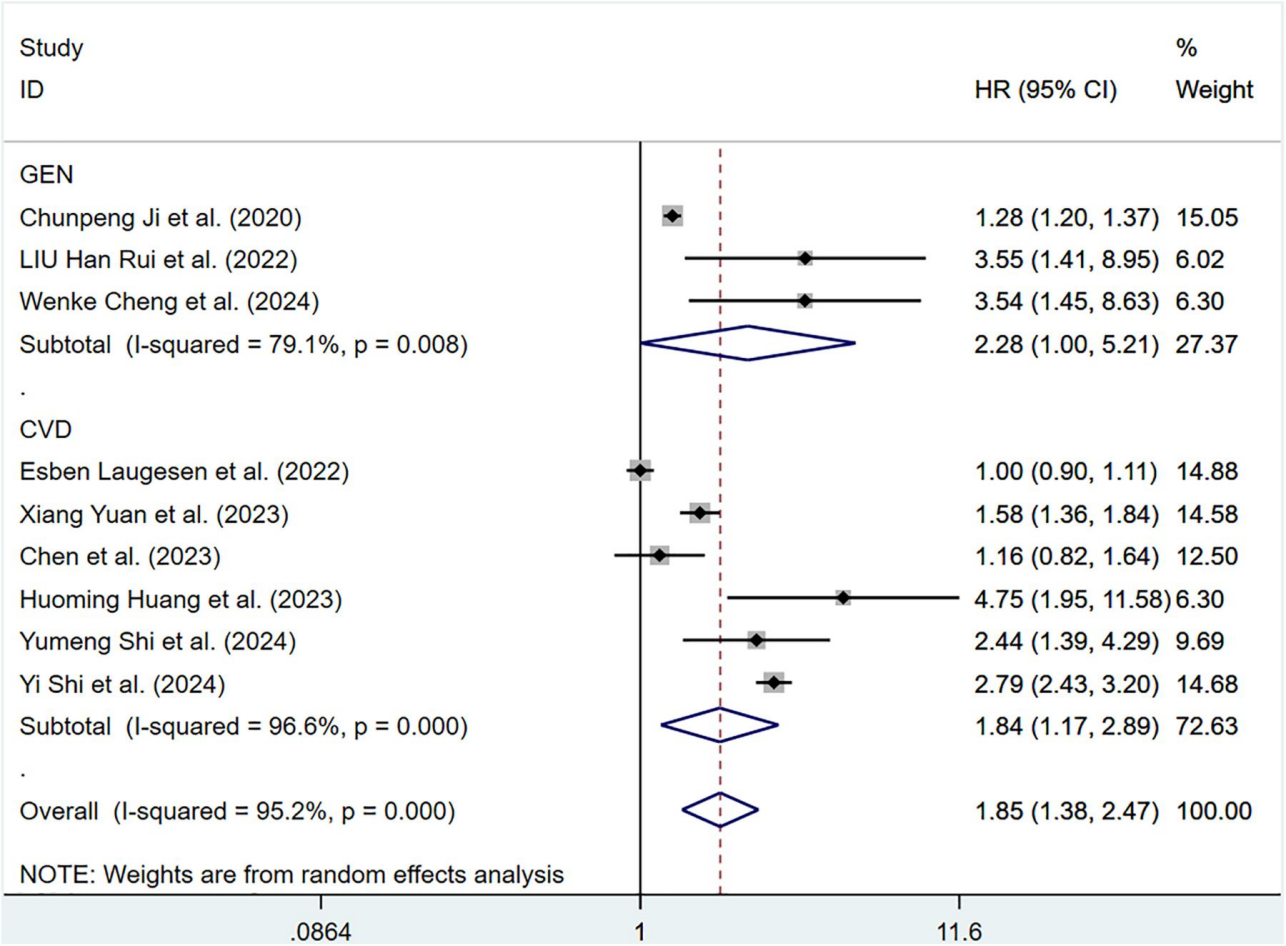
Subgroup analysis of all-cause mortality in general population and those with CVD, analyzed according to categorical variables. The results of each study are represented in point estimates (represented by black diamonds in the figure), and the horizontal line around each point estimate represents the 95% confidence interval (CI) of the research results. The width of the CI represents the accuracy of the estimated value; a narrower CI means higher accuracy. The gray square represents the effect size weight of a single study, and its area is proportional to the weight of the study in the meta-analysis. The hollow diamond at the bottom represents the summary effect size and its CI, which represents the final result of the meta-analysis. The coincidence point between the center position of the diamond and the red dashed line is the summary effect size. The horizontal axis (*X*-axis) represents the numerical range of the effect size. The logarithmic scale centered around 1 in the figure represents negative effect if it is less than 1, and positive effect if it is greater than 1. The black solid line crossing vertically through 1 represents “no effect” or “no difference”. If the CI of the study intersects with the invalid line, it indicates no statistical significance, otherwise it can be considered as significant differences.

Twelve cohort articles involving 311,868 participants reported the correlation of ePWV with all-cause mortality. Compared with individuals with low ePWV, individuals with high ePWV showed a significantly higher risk of all-cause mortality (HR = 1.85, 95%CI, 1.38–2.47) (Supplementary Figure S6). Heterogeneity analysis showed considerable heterogeneity among these studies (*P* < 0.001, *I*^2^ = 95.2%). Therefore, a random-effects model was applied. Analyses of population types verified that individuals with high ePWV had significantly higher risk levels in both the general population group (HR: 2.28, 95%CI: 1.00–5.21, *P* = 0.008, *I*^2^ = 79.1%) and the CVD group (HR: 1.84, 95%CI: 1.17–2.89, *P* < 0.001, *I*^2^ = 96.6%) (Figure 6). This result indicated that the population type was not a possible source of heterogeneity. Further investigation revealed a significant reduction in intergroup heterogeneity in studies enrolling patients with hypertension (*P* = 0.651, *I*^2^ = 0.0%), suggesting that the type of underlying comorbidities may be a source of heterogeneity (Supplementary Table S5).

**Figure 7:**
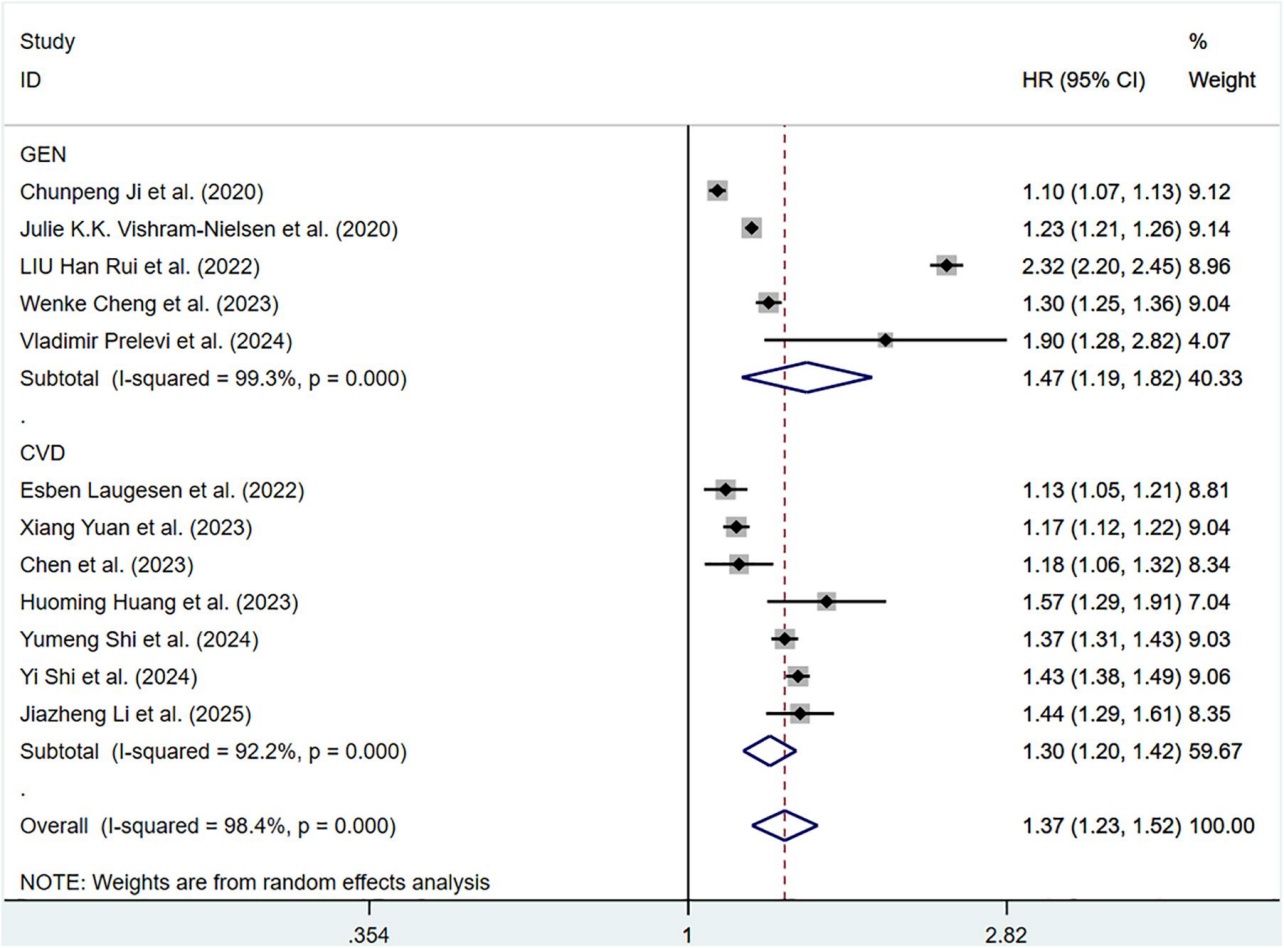
Subgroup analysis of all-cause mortality in general population and those with CVD, analyzed according to continuous variables. The results of each study are represented in point estimates (represented by black diamonds in the figure), and the horizontal line around each point estimate represents the 95% confidence interval (CI) of the research results. The width of the CI represents the accuracy of the estimated value; a narrower CI means higher accuracy. The gray square represents the effect size weight of a single study, and its area is proportional to the weight of the study in the meta-analysis. The hollow diamond at the bottom represents the summary effect size and its CI, which represents the final result of the meta-analysis. The coincidence point between the center position of the diamond and the red dashed line is the summary effect size. The horizontal axis (*X*-axis) represents the numerical range of the effect size. The logarithmic scale centered around 1 in the figure represents negative effect if it is less than 1, and positive effect if it is greater than 1. The black solid line crossing vertically through 1 represents “no effect” or “no difference”. If the CI of the study intersects with the invalid line, it indicates no statistical significance, otherwise it can be considered as significant differences.

The pooled HR of all-cause mortality with an elevation of 1 m/S and 1 SD in ePWV was 1.37 (95%CI: 1.23–1.52) (Supplementary Figure S7). Considerable heterogeneity was observed among these studies (*P* < 0.001, *I*^2^ = 98.4%). Thus, a random-effects model was adopted. Individuals with high ePWV had significantly higher risk levels in both the general population group (HR: 1.47, 95%CI: 1.19–1.82, *P* < 0.001, *I*^2^ = 99.3%) and the CVD group (HR: 1.30, 95%CI: 1.20–1.42, *P* < 0.001, *I*^2^ = 92.2%) (Figure 7). The population difference was not a source of heterogeneity. When analyses were restricted to studies involving a well-defined population with impaired cardiac function, a significant reduction in heterogeneity was observed (*P* = 0.682, *I*^2^ = 0.0%), suggesting that cardiac function status may be a contributing factor to the overall heterogeneity (Supplementary Table S5).

### Sensitivity analyses

Sensitivity analyses were conducted on all-cause mortality and total CV events, and the impact of studies on the pooled results was assessed through a case-by-case exclusion method. The results indicated that excluding individual studies in sequence did not have a significant impact on all pooled outcomes. This indicated that there was a certain degree of stability in the results of this meta-analysis. The results of the sensitivity analysis are shown in (Supplementary Figure S8–S12).

### Publication bias

Funnel plots visually demonstrated evidence of publication bias in studies exploring the association between ePWV and various outcomes, including total CV events, all-cause mortality, and CV mortality. Studies investigating the association between ePWV and total CV events exhibited significant publication bias, regardless of whether the outcome was analyzed as a categorical or continuous variable. Egger's test confirmed these findings, with *P* = 0.001 for the categorical variable and *P* = 0.014 for the continuous variable. Therefore, the trim-and-fill method was employed to supplement the analysis. In terms of categorical variables, three studies were added, resulting in a combined effect size adjustment from HR = 1.85 (95%CI: 1.38–2.47) to HR = 1.899 (95%CI: 1.516–2.378). For continuous variables, five iterations were performed to supplement five additional studies, adjusting the HR from 1.36 (95%CI: 1.27–1.45) to 1.287 (95%CI: 1.212–1.367). The slight discrepancy observed between the initial and adjusted results indicates that, despite the potential for publication bias, its influence on the overall findings is likely negligible. In the study of the continuous variable relationship between ePWV and all-cause mortality, although the funnel plot suggests the presence of bias, the Egger test result (*P* = 0.298) indicates that the bias is not statistically significant. For studies examining ePWV in relation to all-cause or CV mortality (categorical variables) and ePWV in relation to CV mortality (continuous variables), Egger's test and publication bias analyses were not conducted due to the small number of included studies (*n* < 10 or *n* < 5). This approach was consistent with guideline recommendations and pre-specified standards (Supplementary Figure S13–S17).

## Discussion

This research systematically reviewed and performed a meta-analysis of studies assessing the correlation between ePWV and clinical outcomes. In general, higher ePWV was significantly correlated with an increased rate of CV events and all-cause mortality. Additionally, the research results found that the predictive value of ePWV showed some dependency on the target population, differing from its overall association. Population analysis demonstrated that ePWV had different effects on adverse outcomes in the general population compared to those with CVD. In predicting total CV events, elevated ePWV showed higher predictive ability in CVD patients. This indicated that in individuals with existing cardiovascular damage, the arterial stiffness reflected by ePWV may be a key factor in the recurrence or progression of adverse events. Regarding all-cause mortality, the correlation with ePWV was more significant in the general population compared to CVD patients. This suggested that abnormal elevation of ePWV not only signals CV risk but is also likely linked to an increased risk of mortality from other diseases, demonstrating that ePWV may have a more extensive predictive capability. In summary, for patients with CVD, ePWV can be utilized as an important tool for risk stratification; whereas, in the general population, ePWV serves as a critical indicator for screening individuals at high risk for cardiovascular and other adverse events. Thus, rational application of ePWV according to the type of population may provide clinical guidance for managing cardiovascular and mortality risks.

Our meta-analysis results support ePWV as a potential biomarker for predicting CV and mortality. Actively monitoring ePWV levels and improving them through lifestyle or medication can help predict and design future interventions for relevant populations. Vlachopoulos et al. found in the SPRINT study population that ePWV predicted endpoints such as acute coronary syndrome, stroke, heart failure, or all-cause death in patients, with a primary CV outcome HR of 1.30 (95%CI: 1.17–1.43; *P* < 0.001) and an all-cause mortality HR of 1.65 (95%CI: 1.46–1.86; *P* < 0.001). The anticipated outcomes exhibited no association with the Framingham risk score, and its predictive ability was better than FRS regarding all-cause mortality or major CV outcomes, indicating that ePWV was gradually becoming a new biomarker for CV risk prediction (40).

The possible mechanisms by which ePWV predicts CV events and mortality risk can be explored based on current analyses. It is widely known that the aging of the vascular system results in structural and functional changes, mainly including an increase in vascular thickness and hardness, and cfPWV has long been used as a reference indicator for vascular aging. Kevin S. Heffernan et al. undertook a study to assess the efficacy of ePWV, demonstrating that the correlation of ePWV with other established vascular aging indicators, including carotid intima-media thickness (cIMT), carotid stiffness and carotid enhancement index (cAIx) measured by elasticity modulus (cEp), was greater than that of cfPWV with these indicators, supporting the establishment of ePWV as an effective measure of vascular aging (41). cfPWV serves as the primary method for assessing arterial stiffness, with numerous studies even considering ePWV as an alternative standard to traditional risk assessments such as cfPWV to evaluate arterial stiffness. The mechanism of its predictive value can be explained by cfPWV, which infers the association between arterial stiffness and CV physiological and pathological changes. Arterial stiffness is mainly manifested as arterial dilation, hypertrophy, degenerative changes in the media layer of the arteries, arterial wall stiffness, and decreased buffering force, which may result in coronary perfusion disorders. Clinically, there is often a disproportionate increase in systolic blood pressure and pulse pressure, leading to clinical events such as CVD and CV death (42, 43).

ePWV is derived from equations related to age and blood pressure. This estimated index compensates for the shortcomings of equipment requirements and measurement conditions, and reflects the complex correlation of age, blood pressure, and vascular aging with arterial stiffness. In studies measuring age and blood pressure, ePWV had insight into vascular aging that other measurement indicators (such as cfPWV) did not possess (44). In most of the included studies, patients with high ePWV might have older age, higher blood pressure, and higher weight. We believed that patients with high ePWV might have a higher baseline risk than those with low ePWV. However, all the studies we included adjusted for potential confounding factors among patients, and most studies showed significant predictive effects of ePWV even after statistical adjustments for the components of ePWV (age, square of age, and systolic and diastolic blood pressure), demonstrating that ePWV was not just a simple measure of age and blood pressure for predicting risk. The potential complex interactions between age and blood pressure have been revealed, which may not have been fully covered by traditional risk scores and cfPWV. In other words, we may have underestimated the evaluative role of age and blood pressure. Since arterial stiffness often accompanies adverse reactions such as elevated blood pressure and other risk factors, it has long been taken as a complication of hypertension. However, recent studies have shown that stiffness can occur prior to the pathogenesis of hypertension and accelerate its further development. Hypertension can cause arterial stiffness, and an increase in arterial pressure can lead to the destruction of elastic fiber structures in the arterial wall, resulting in a decrease in elasticity and making the arteries even stiffer. Over time, the ability of arteries to regulate blood flow is impaired, thus exacerbating hypertension and forming a bidirectional feedback loop (45–47). In a meta-analysis, consistent correlations were observed. For every 20 mmHg increase in SBP, cfPWV increased by 1.14 or 0.94 m/s every decade, resulting in an approximately 15% higher all-cause mortality rate (45). The results of a cohort study based on 54,849 individuals conducted by Haojia Chen's team showed that ePWV was positively correlated with both mean systolic and mean diastolic blood pressure in general population, and the risk of hypertension increased with the elevation of ePWV (48). The influence of blood pressure and age is synergistic, and the elastic arteries near the heart are extremely sensitive to the effects of blood pressure and age, which are crucial determinants of arterial stiffness. It seems that ePWV is more dependent on these two parameters, but like PWV, ePWV has been proven to be independent of traditional risk scores and factors (such as FRS and SCORE) to predict future CV and mortality events (6, 49).

There is currently no established standard cutoff value for ePWV to predict specific outcomes for specific populations. However, based on existing research evidence, the establishment of predictive reference values for ePWV is gaining increasing interest. According to Vishram-Nielsen JKK et al., the general population with a moderate SCORE and ePWV ≥ 9.4 m/s showed the highest rates of all-cause mortality, CV mortality, and the risk of combined CV events (24). Kim BS et al. found that the optimal cutoff level for ePWV in predicting CV events in middle-aged individuals from a time-dependent ROC curve analysis is approximately between 8.82 and 10.08 m/s (26). Li D et al. applied a two-stage linear regression model to evaluate the non-linear correlation of ePWV with the risk of all-cause mortality and CVD mortality (non-linear *P* < 0.001). When the ePWV values fell within the range of 6.7–8.7 m/s and 7.2–8.5 m/s, the growth curves of all-cause mortality and CVD mortality were steeper. Once ePWV surpassed 8.7 m/s and 8.5 m/s, the growth curves for all-cause mortality and CVD mortality plateaued, suggesting that the risk of all-cause mortality escalation was more rapid before reaching the elevated threshold (50). Chunwei Chen et al. adopted Maxstat and ROC curves to calculate the optimal cut-off value for evaluating all-cause mortality in individuals with coronary heart disease, which was 11.15 m/s. When ePWV ≤ 11.15 m/s, the mortality was lower (HR < 1), but when ePWV>11.15 m/s, the risk of all-cause mortality showed a significant linear increase (36). Wenke Cheng et al. found that the risk of all-cause mortality at 1 m/s was twice as high in hypertensive patients with ePWV≥13.36 m/s compared to those with ePWV<13.36 m/s. This inflection point should be highly valued in clinical practice. If a value exceeds this point, the risk of all-cause mortality will sharply increase (51). It is worth noting that the value of ePWV does not increase synchronously with the estimated effect. For example, the study by Huang H reported a non-linear relationship between ePWV and CV death. In stroke patients with ePWV ≥ 12.1 m/s, an increase of 1 m/s in ePWV was not associated with predictive value for CVD and mortality risk (HR = 0.99) (17). Compared to the general population, ePWV has a higher predictive ability for overall CV events and all-cause death in CVD patients. This may be a “selection” phenomenon, where subjects with a history of CVD have poorer arterial structure and function, making them more prone to adverse CV events such as CV death. ePWV can be calculated using readily available clinical data and a simple formula, obviating the need for dedicated equipment. This simplifies the assessment process and provides a practical alternative for primary healthcare facilities with limited resources and lack of specialized equipment, thus addressing the limitations of direct measurement. ePWV offers improved accessibility in CV risk assessment and can be utilized as a rapid initial screening tool to identify high-risk populations, thereby informing further interventions or precise evaluations. Due to its convenient calculation, ePWV can be readily implemented in large cohort studies, enabling the investigation of the association between arterial stiffness and diverse diseases, thus promoting epidemiological research. In the management of high-risk patients, the combined assessment of arterial stiffness using directly measured PWV and ePWV can enhance the robustness of evaluation findings (52–54).

Although many studies have been published to support the predictive role of ePWV, this meta-analysis is the inaugural study to furnish the most recent and consolidated assessments of ePWV. Firstly, a detailed search strategy was developed, which enabled us to capture more relevant research. Inevitably, certain limitations exist in this study. Firstly, the analysis results show significant heterogeneity. Given the limited number of studies included and inadequacies in reporting pertinent details, we are unable to explore heterogeneity sources more accurately through subgroup analysis and meta-regression analysis, which may reduce the certainty of evidence and recommendations. Further research is needed to confirm this. Secondly, although ePWV is derived based on age and MBP, the cutoff values of ePWV in most studies are slightly different. In the included studies, the critical value separating high and low ePWV was found to be around 10 m/s. However, risk stratification based on specific ePWV values is challenging, which may be related to specific circumstances in different studies, resulting in a certain degree of cohort dependence in defining these thresholds. Furthermore, the limited number of original studies restricts a more extensive use of ePWV values for meta-regression analysis. These limitations may contribute to biases or significant heterogeneity in research findings. It is anticipated that more relevant studies will emerge in the future to investigate and standardize the critical values. Thirdly, the majority of the included samples come from Asian populations, with relatively few from other regions such as Europe and the Americas. This uneven regional distribution may affect the generalisability of the results. Especially, differences in race, culture, and healthcare systems may lead to regional heterogeneity in the effect size, and unevenly distributed populations may result in lower statistical efficacy in some regions. Therefore, future research should focus on further exploration from different geographical regions. Fourthly, limiting the analysis to studies published in English may introduce selection bias. Therefore, we look forward to more researchers from diverse regions incorporating a broader range of studies. Moreover, small-scale studies may have small study effects and may also lead to asymmetric funnel plots.

## Conclusion

ePWV emerges as a robust predictor of CV events, CV mortality, and all-cause mortality. With the escalation of ePWV, individuals with CVD are at a heightened risk of encountering total CV events, and in cases of high ePWV, the general population is more sensitive to the occurrence of all-cause death. These findings support the application of ePWV in clinical practice. However, given the existing limitations, prospective, extensive, and meticulously crafted studies are needed to reinforce the predictive capacity of ePWV and define reference values more conclusively.

## Data Availability

The original contributions presented in the study are included in the article/Supplementary Material, further inquiries can be directed to the corresponding author.
